# Exploring Three Core Psychological Elements When Treating Adolescents on the Autism Spectrum: Self-Awareness, Gender Identity, and Sexuality

**DOI:** 10.7759/cureus.14130

**Published:** 2021-03-26

**Authors:** Ann Genovese

**Affiliations:** 1 Psychiatry and Behavioral Sciences, University of Kansas Health System, Kansas City, USA

**Keywords:** autism, sexuality, adolescence, self-awareness, gender identity, psychological

## Abstract

For those growing up on the autism spectrum, adolescence is associated with unique challenges. This narrative review explores three core psychological elements for clinicians to consider when treating adolescents on the autism spectrum: self-awareness, gender identity, and sexuality. Developmental tasks of adolescence include adaptation to a maturing mind and body, increased expectations for independence, and the ability to establish satisfying interpersonal relationships. What are welcome opportunities for non-autistic peers can become nearly insurmountable hurdles for autistic teens, which, in turn, could lead to crisis, particularly if skills needed for success in managing these tasks have not yet been acquired.

## Introduction and background

Adolescence is the span in an individual’s life when one transitions from life as a child to that of an adult. For the adolescent on the autism spectrum, the anticipated challenges associated with adolescence are compounded by core symptoms associated with autism spectrum disorder (ASD), which include functional impairments in social and communication skills as well as idiosyncratic or repetitive patterns of behavior, activities, or interests [1].

The World Health Organization (WHO) states that adolescence is between 10 and 19 years of age, during which the young person is expected to incorporate the knowledge and capability of managing emotions and relationships, acquire the skills necessary to navigate the adolescent years, and prepare for adult roles [2]. The American Academy of Pediatrics defines the span of adolescence from 11 to 21 years of age [3], whereas the Canadian Paediatric Society rejects the notion that adolescence should be based on chronological age, favoring instead a functional definition determined by the biopsychosocial readiness of young people to enter adulthood [4].

Three core psychological elements to consider when treating adolescents on the autism spectrum are self-awareness, gender identity, and sexuality. The developmental tasks of the adolescent include adjustment to the onset of puberty, the completion of growth, assuming a sexually mature body, expanding cognitive abilities, achieving a greater degree of independence, and establishing a clearer sense of personal identity [5]. Identity is defined as a person’s sense of ‘self’ defined by physical, psychological, and interpersonal characteristics that is not in whole shared with another person, as well as a range of affiliations, including ethnic, cultural, and social roles [6].

The task of identity development can be even more difficult for autistic teens [7]. Autistic adolescents often perceive themselves as less socially or physically competent and tend to endorse a lower sense of self-worth compared to non-autistic peers [8]. Autistic teens may end up assuming a negative self-concept based on peers’ perceptions of their being ‘different’ [9]. These challenges are compounded when autistic youth are impacted by psychological and/or psychiatric disorders, which are more common in autistic than non-autistic cohorts [10] and often persist over the course of their lifespan [11].

Additional challenges in adolescence occur as social expectations mount and relationships become more complex. The desire for social relationships, which generally increases during adolescence, can be impeded due to social difficulties including the risk for rejection of autistic youth by peers [12]. While in the process of forming their personal identity, autistic adolescents are developing their shared social identity, often perceiving themselves in a minority group status compared to their non-autistic peers [13].

Adolescents who identify as a member of the autistic community develop a shared identity and group membership, which provides a sense of self-understanding, belonging, and purpose [14]. A shared social identity can protect adolescents against the negative consequences of minority group status by giving them a sense of support, thus buffering the effects of discrimination [15].

The neurodiversity movement rejects the notion that autism is a ‘disorder’ per se and instead promotes autism as a positive identity associated with unique personal strengths and abilities [16]. While many autistic adolescents have learned to embrace autism as part of ‘who they are’ and take pride in their autistic identity [17], not all young people with autism self-identify as autistic or see themselves as being part of the autistic culture [18].

## Review

Self-awareness and self-regulation

Self-Awareness

Autism originates from the Greek word ‘autos’, which translates literally to ‘self’. Kanner describes differences in the understanding of the self in his original case descriptions of children with ASD [19]. Subsequent descriptions from clinical case reports, autobiographies, self, and parent reports similarly conclude that differences in self-awareness likely relate to core features of autism [20].

Self-awareness refers to experiencing oneself as ‘the object of one’s own attention’ and cognizant of one’s own mental states, including perceptions, attitudes, intentions, emotions, and behaviors [21]. Factors that may impact self-awareness include common autism-related impairments including an incomplete understanding of the thoughts and feelings of others, difficulty in interpreting their own or others’ perspectives and preferences, and limited ability to adjust their own behaviors to adapt and conform within social contexts [22].

Self-Regulation

Self-awareness provides the foundation for the development of self-regulation, including emotion regulation. Inner speech, or internalized self-talk, in autistic individuals tends to be atypical compared to non-autistic peers [23]. One’s ability to ‘think in speech’ is a critical component of emotional and behavioral self-regulation, and therefore impairment in inner speech, which is commonly associated with autism, leads to difficulty in self-regulating in interpersonal relationships [24].

Emotion regulation is a complex process that involves the monitoring and adjustment of emotional responses, occurs either intentionally or automatically, and helps maintain or modify the type, intensity, and duration of emotions [25]. The process of emotion regulation involves the ability to identify one’s emotions, determine whether they need to be regulated, and implement strategies to modulate these emotions when needed [26]. Emotion dysregulation occurs when emotions are not effectively regulated and can manifest as negative mood states or excessive irritability, which are common in autism and associated with a wide range of negative outcomes in general physical and psychological health [27].

Gender and sexuality

Gender Differences in Autism

A male-to-female sex ratio of about 4:1 across the autism spectrum has been consistently demonstrated for many years in community‐based studies, for reasons not entirely understood [28]. Studies suggest that core symptoms and impairments related to autism may differ according to gender. Autistic boys generally show greater impairment in social skills and have higher rates of restrictive and repetitive behaviors compared to autistic girls [29,30]. As a group, females on the autism spectrum compared to males are reported to have more problems with daily living skills and executive functioning, a finding supported in studies controlling for ASD symptom severity, intelligence, and social communication skills [31].

Patterns of social and behavioral adaptation and psychopathology that commonly occur in the context of autism can be very different for boys than for girls. Autistic males are reported by their teachers to have more externalizing (acting out behaviors) and interpersonal problems, whereas autistic females are reported by their parents to have a greater severity of emotional difficulties including depression and anxiety disorders [32,33]. Given similar levels of autistic symptoms, females are less likely to be diagnosed with ASD than males [34], and females on average are diagnosed with autism at a later age than males [35], which may be in part accounted for by the perception that the female phenotype is sometimes not viewed as a ‘typical’ autism presentation, and therefore the referral for an autism diagnostic evaluation is often delayed [36].

Gender Identity

Gender refers to mannerisms, behaviors, and attitudes that when judged in the context of a given era and culture are considered either typically male or female, whereas gender identity is the person’s own sense of their gender, which may or may not correlate with their assigned birth sex. ‘Gender dysphoria’ is a term that applies to individuals who experience incongruence between their assigned sex and gender identity. Rates of gender dysphoria reported by autistic individuals are significantly higher than reported in the general population [37].

Among autistic adolescents and adults, more women than men report gender non-conforming attitudes and emotions. Autistic females are prone to lower social affiliation with a gender group and greater variability in their gender expression, reporting fewer typically feminine and more traditionally masculine traits when compared to autistic males [38]. While gender dysphoria, in general, is relatively rare, the prevalence of autistic features in people referred to gender clinics is comparatively common [39].

Gender diversity refers to the divergence of an individual's gender identity or expression from cultural norms expected for individuals of a given sex. Transgender denotes a person whose gender identity does not correspond with their birth sex [40]. It has been shown that autistic females are more likely to identify with a transgender identity than autistic males and non-autistic female peers [41,42]. Autistic gender-diverse adolescents often have difficulty in self-expression because of autism-related social and communication challenges compounded by the fear of authentic gender expression due to perceived negative bias and discrimination against transgender people [43].

Clinical guidelines developed for adolescents with autism and gender dysphoria state that autistic adolescents are capable of gender self-determination [44]. Furthermore, the World Professional Association for Transgender Health (WPATH) Standards of Care for the Health of Transsexual, Transgender, and Gender Nonconforming People assert that co-occurring conditions should not exclude individuals from gender-affirming care [45].

Sexuality

Sexual health refers to physical, social, and psychological well-being in the context of sexuality. It is premised upon a positive and respectful approach to sexuality and the possibility of having pleasurable and safe sexual experiences, free of discrimination, coercion, or violence [2]. The literature reveals that individuals on the autism spectrum have fewer opportunities for appropriate informal and formal sexual health education, which leaves them at a disadvantage from peers who receive this information [39]. Unfortunately, sexual harassment and assault are reported to impact autistic women at a disproportionate rate. Compared to general population peers, a greater proportion of autistic women report having experienced unwanted sexual encounters compared to non-autistic women [42].

Sexuality is a core aspect of the human experience at all stages of life and incorporates gender identity, sexual orientation, eroticism, pleasure, intimacy, sexual experiences, and reproduction [2]. Sexuality is experienced in thoughts, desires, attitudes, behaviors, roles, and relationships. Sexual behaviors can be expressed and directed toward either the self or another. Experience with solo and partnered sexuality and with romantic relationships is common for most adolescents and adults on the autism spectrum [46].

Sexual orientation defines the experience of intimate sexual attraction to someone of the same sex, other sex, or either sex. Some studies find similar proportions of same-sex attraction or experience among autistic participants and non-autistic peers, which is typically around 5-10% [47], whereas other studies report higher levels of non-heterosexual feelings and experiences in autistic adolescents and adults [48]. More autistic women compared to autistic men identify as bi-sexual, endorsing sexual attraction to both same- and opposite-sex partners [49].

## Conclusions

Three core psychological elements to consider when treating adolescents on the autism spectrum are self-awareness, gender identity, and sexuality. The developmental tasks of adolescence include exploration and consolidation of one’s sense of identity. Self-awareness and self-regulation are fundamental in the process of emotional maturation. The work involved in solidifying a stable sense of self is shared by all adolescents, but the process can be complicated by challenges commonly faced by autistic youth.

It is important to recognize the interactions of psychological and emotional development with pubertal maturation. Autistic young people struggle with gender identity and sexuality at least as often, and likely more than other teens, and therefore professionals who interface with these adolescents need to be informed and aware so that concerns can be explored, safety can be assessed, and additional resources can be provided when needed.

The limitations of this narrative review include the relative dearth of evidence-based literature investigating these specific topics. Additional studies exploring longer term outcomes for autistic young people who are struggling with their gender identity or sexuality would help determine best psychotherapeutic practices.
